# Cilantro microbiome before and after nonselective pre-enrichment for *Salmonella* using 16S rRNA and metagenomic sequencing

**DOI:** 10.1186/s12866-015-0497-2

**Published:** 2015-08-12

**Authors:** Karen G. Jarvis, James R. White, Christopher J. Grim, Laura Ewing, Andrea R. Ottesen, Junia Jean-Gilles Beaubrun, James B. Pettengill, Eric Brown, Darcy E. Hanes

**Affiliations:** U. S. Food and Drug Administration, Center for Food Safety and Applied Nutrition, OARSA, Laurel, MD USA; Oak Ridge Institute for Science and Technology, Oak Ridge, TN USA; U. S. Food and Drug Administration, Center for Food Safety and Applied Nutrition, ORS, College Park, MD USA

## Abstract

**Background:**

*Salmonella enterica* is a common cause of foodborne gastroenteritis in the United States and is associated with outbreaks in fresh produce such as cilantro. *Salmonella* culture-based detection methods are complex and time consuming, and improvments to increase detection sensitivity will benefit consumers. In this study, we used 16S rRNA sequencing to determine the microbiome of cilantro. We also investigated changes to the microbial community prior to and after a 24-hour nonselective pre-enrichment culture step commonly used by laboratory analysts to resuscitate microorganisms in foods suspected of contamination with pathogens. Cilantro samples were processed for *Salmonella* detection according to the method in the United States Food and Drug Administration Bacteriological Analytical Manual. Genomic DNA was extracted from culture supernatants prior to and after a 24-hour nonselective pre-enrichment step and 454 pyrosequencing was performed on 16S rRNA amplicon libraries. A database of Enterobacteriaceae 16S rRNA sequences was created, and used to screen the libraries for *Salmonella*, as some samples were known to be culture positive. Additionally, culture positive cilantro samples were examined for the presence of *Salmonella* using shotgun metagenomics on the Illumina MiSeq.

**Results:**

Time zero uncultured samples had an abundance of Proteobacteria while the 24-hour enriched samples were composed mostly of Gram-positive Firmicutes. Shotgun metagenomic sequencing of *Salmonella* culture positive cilantro samples revealed variable degrees of *Salmonella* contamination among the sequenced samples.

**Conclusions:**

Our cilantro study demonstrates the use of high-throughput sequencing to reveal the microbiome of cilantro, and how the microbiome changes during the culture-based protocols employed by food safety laboratories to detect foodborne pathogens. Finding that culturing the cilantro shifts the microbiome to a predominance of Firmicutes suggests that changing our culture-based methods will improve detection sensitivity for foodborne enteric pathogens.

**Electronic supplementary material:**

The online version of this article (doi:10.1186/s12866-015-0497-2) contains supplementary material, which is available to authorized users.

## Background

Cilantro, like many leafy green vegetables that are available year round and usually consumed raw, is difficult to clean and therefore a possible vehicle for transmission of enteropathogenic bacteria. Cilantro has been the target of multiple recalls due to *Salmonella* contamination over the last decade and in 1999 an outbreak of *Salmonella* serotype Thompson was linked to cilantro used to prepare salsa at restaurants in California [1–3].

The Food and Drug Administration (FDA) Bacteriological Analytical Manual (BAM) method for the detection of *Salmonella* in cilantro involves a 24-hour nonselective pre-enrichment step followed by two parallel selective 24-hour enrichments in Rappaport-Vassiliadis and Tetrathionate Broths, and plating on differential/selective agars (http://www.fda.gov/Food/FoodScienceResearch/LaboratoryMethods/ucm070149.htm). Improvements to decrease the time to detect foodborne pathogens are economically desirable to the FDA, especially during outbreaks when reducing exposure to contaminated food in the general population is a primary concern.

Recent advances in DNA sequencing technology have reduced costs and time to results making culture independent high-throughput sequencing technologies more accessible to many laboratories. Shotgun metagenomic sequencing of the microbiomes as well as 16S rRNA amplicon studies have been used to characterize microbial communities in foods to identify spoilage associated, pathogenic, and beneficial organisms [4, 5]. For example, a 16S rRNA amplicon study on Kimchi fermentation to track changes in microbial diversity resulted in an increased understanding of the fermentation process, which has led to improved production methods [6]. Metagenomic sequencing also identified a novel fish pathogen *Kudoa septempunctata*, as the causative agent of food poisoning during a Japanese outbreak traced back to the consumption of raw fish [7]. 16S rRNA analysis of Latin-style and artisanal cheeses have revealed significant differences in the bacterial composition among different brands of the same type of cheese, and identified microbes not previously associated with particular types of cheese, revealing how the raw materials and preparation methods used during cheese fermentation can impact changes in microflora [8, 9]. Improving our ability to quickly identify foodborne pathogens using high-throughput sequencing has been explored, for example, in tomato fruit and plants, with the goal of identifying co-enriched organisms when FDA BAM methods were employed [10, 11].

In this study, we utilized 16S rRNA sequencing to characterize the changes to the cilantro microbiome during a nonselective pre-enrichment in broth culture. The primary goal of our cilantro study was to determine the baseline population of microbes colonizing cilantro, and then to assess community composition shifts during the initial culturing steps used by the FDA BAM to detect enteric pathogens in contaminated leafy greens. Additionally, we sought to identify *Salmonella* specific 16S rRNA gene signatures in culture positive cilantro samples using a newly developed 16S rRNA database specific to Enterobacteriaceae as well as BLASTn and MetaPhlAn analysis of shotgun metagenomes.

## Results

### Sequencing results

Cilantro samples were provided through the United States Department of Agriculture Microbiological Data Program from various distribution centers throughout the United States from July to December 2011 and April to October 2012 (See Additional file 1) [12]. Nine cilantro samples were culture-positive for *Salmonella*. Thirty-five time-zero (T0) and 56 24-hour pre-enrichment (T24) cilantro samples were sequenced using 16S 454 pyrosequencing generating 354,019 quality filtered bacterial 16S rRNA gene sequences. The average number of reads per sample was 2,860 ± 2,557 and 4,534 ± 2,526 in the T0 and T24 samples, respectively (see Additional file 2). The non-specific amplification of 18S rRNA sequences resulting in excess chloroplast contaminant associated with cilantro material (40.1 % in T0 vs. 0.4 % in T24) caused the relatively low level of bacterial 16S rRNA sequence reads per sample in the T0 group (see Additional file 2).

### Cilantro microbiome

**Fig. 1:**
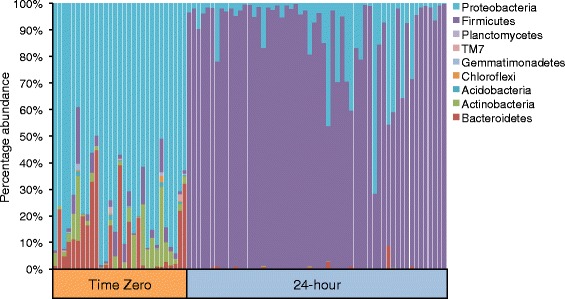
Phyla representing at least 1 % of the total abundances in the cilantro microbiome (T0) and after 24-hour nonselective mBPW pre-enrichment (T24)

Quantitative Insights into Microbial Ecology (QIIME) [13] analysis of cilantro 16S rRNA gene sequences revealed a microbiome (T0 cilantro samples, Fig. 1) comprised mainly of Proteobacteria (77 %), followed by Bacteroidetes (12 %), Actinobacteria (6 %) and Firmicutes (4 %).

**Fig. 2:**
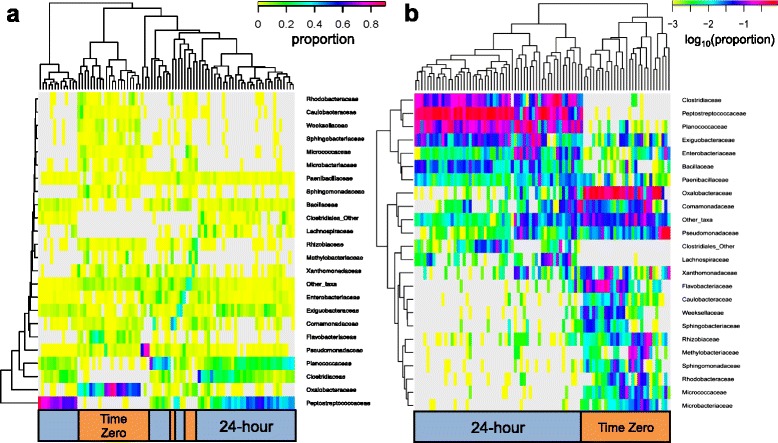
Unsupervised hierarchical clustering of samples using family level profiles. Values reflect (**a**) proportions and (**b**) log-normalized proportions (e.g. -1 ~ 10 %, −2 ~ 1 %, −3 ~ 0.1 %) to increase the weighting of low frequency members. Utilizing the log-normalized profiles, we find distinct clustering of T0 and T24 samples. Dendrograms were generated using a Euclidean distance metric with furthest neighbor clustering

Further characterization of the microbiome at the family level using an unsupervised hierarchical clustering approach resulted in multiple clades of Proteobacteria within the T0 samples (Fig. 2a). The largest clade consists of Oxalobacteraceae, known colonizers of the rhizosphere and roots of many plant species (Fig. 2a) [14]. Other Proteobacteria present in high abundance within the cilantro microbiome include members of the Comamonadaceae and Pseudomonadaceae (Fig. 2a and b).

### The effect of a 24-hour nonselective pre-enrichment

Culturing cilantro for 24-hours in a nonselective pre-enrichment broth used for resuscitation of microorganisms in foods called, modified Buffered Peptone Water (mBPW), resulted in a dramatic shift in community members to a predominance of Firmicutes and then Proteobacteria (Fig. 1). Unsupervised hierarchical clustering of proportional abundances at the family level revealed multiple clades within the T24 samples, the two largest consisting of Peptostreptococcaceae and Clostridiaceae, and a third comprised mainly of Planococcaceae all members of the Firmicutes (Fig. 2a). The relative abundances of family level taxa were then log-normalized prior to unsupervised hierarchical clustering of samples and taxa using a Euclidean distance metric with complete linkage methodology. Heatmap values, reflecting log-normalized proportions (i.e. -1 ~ 10 %, −2 ~ 1 %, −3 ~ 0.1 %), show two subgroups clearly distinguished by enrichment state (T0 or T24) (Fig. 2b). Family members present at very low levels in the microbiome (T0 samples), including Clostridiaceae, Peptostreptococcaceae, and Lachnospiraceae, were highly enriched after 24-hours (Fig. 2b). The Enterobacteriaceae, Bacillaceae, and Paenibacillaceae also appear to increase in abundance after pre-enrichment, and the proportional abundances of the Gram-negative Bacteroidetes, Proteobacteria, and Gram-positive Actinobacteria were visibly decreased (Fig. 2b).

We analyzed the 16S rRNA proportional abundance data of T0 and T24 samples using MetaStats to determine the significance of the community shifts predicted by QIIME (Table 1) [15]. Considering taxa representing at least 0.5 % of the total population in the cilantro microbiome (T0 samples), the proportional abundance of 18 of 22 taxa significantly decreased following a 24-hour nonselective pre-enrichment (Table 1a). The exceptions were the [Exiguobacteraceae], Enterobacteriaceae, Paenibacillaceae, and Bacillaceae, which all had significant increases in proportional abundance, ranging from 1.5 to 5.0 fold changes (Table 1a). Changes in the cilantro microbiome, induced by pre-enrichment, resulted in 14 taxa being present at or above 0.5 % of the total population (Table 1b). The proportional abundances of Peptostreptococcaceae, Planococcaceae, Clostridiaceae, Lachnospiraceae, and Aeromonadaceae significantly increased by more than a 50 % following 24-hour nonselective pre-enrichment (Table 1b). The change in microbial community composition to a predominance of microaerophilic and anaerobic species suggests a shift toward an oxygen-depleted environment after 24-hours (Table 1).

**Table 1:** MetaStats analysis of relative abundance (>0.5 %)

	%T0	%24-hour	Fold change	*P* value
a.) The most abundant members at time zero				
Oxalobacteraceae	38.63	0.96	−40.21	0.0002
Pseudomonadaceae	10.62	1.36	−7.79	0.0158
Flavobacteriaceae	7.33	0.22	−33.12	0.0002
Comamonadaceae	5.03	2.43	−2.07	0.1982
Xanthomonadaceae	4.11	0.87	−4.72	0.0154
Rhizobiaceae	3.57	0.13	−27.76	0.0002
Methylobacteriaceae	3.50	0.03	−140.14	0.0002
Micrococcaceae	2.52	0.01	−176.45	0.0002
Sphingomonadaceae	2.28	0.03	−79.78	0.0002
Weeksellaceae	2.20	0.07	−33.30	0.0002
Rhodobacteraceae	2.11	0.03	−73.74	0.0002
Microbacteriaceae	2.06	0.02	−104.63	0.0002
[Exiguobacteraceae]	1.84	6.20	3.37	0.0002
Caulobacteraceae	1.58	0.03	−55.16	0.0002
Rickettsiales	1.32	0.01	−92.45	0.0002
Sphingobacteriaceae	1.29	0.08	−17.20	0.0002
Enterobacteriaceae	1.14	3.59	3.13	0.0288
Aurantimonadaceae	1.01	0.01	−93.98	0.0002
Paenibacillaceae	0.99	1.51	1.52	0.1238
Rhizobiales;Other	0.62	0.04	−14.56	0.0002
Bacillaceae	0.61	3.08	5.01	0.0002
Cytophagaceae	0.60	0.01	−111.36	0.0002
b.) The most abundant members after a 24-hour mBPW enrichment				
Peptostreptococcaceae	0.01	40.79	2957.09	0.0002
Planococcaceae	0.31	19.49	62.11	0.0002
Clostridiaceae	0.03	13.54	490.93	0.0002
[Exiguobacteraceae]	1.84	6.20	3.37	0.0002
Enterobacteriaceae	1.14	3.59	3.13	0.0288
Bacillaceae	0.61	3.08	5.01	0.0002
Comamonadaceae	5.03	2.43	−2.07	0.1982
Lachnospiraceae	0.00	1.82	527.18	0.0002
Paenibacillaceae	0.99	1.51	1.52	0.1238
Clostridiales;Other	0.00	1.50	∞	0.0002
Pseudomonadaceae	10.62	1.36	−7.79	0.0158
Oxalobacteraceae	38.63	0.96	−40.21	0.0002
Xanthomonadaceae	4.11	0.87	−4.72	0.0154
Aeromonadaceae	0.00	0.57	164.68	0.0002

The Enterobacteriaceae were also significantly enriched, but not as dramatically as the Firmicutes family members (Table 1). Eighteen Enterobacteriaceae family members were identified in the T0 and T24 samples, and *Serratia, Erwinia, and Trabulsiella* species were the most abundant (see Additional file 3). Moreover, unidentified genera make up 50 and 24 % of the Enterobacteriaceae, in the T0, and T24 samples, respectively (see Additional file 3). It is notable that *Salmonella* was not detected among the Enterobacteriaceae using the QIIME RDP classifier [16] trained on the GreenGenes 16S rRNA database (v13_8) [17, 18], even in the *Salmonella* culture positive samples (see Additional file 3).

### Taxon diversity

**Fig. 3:**
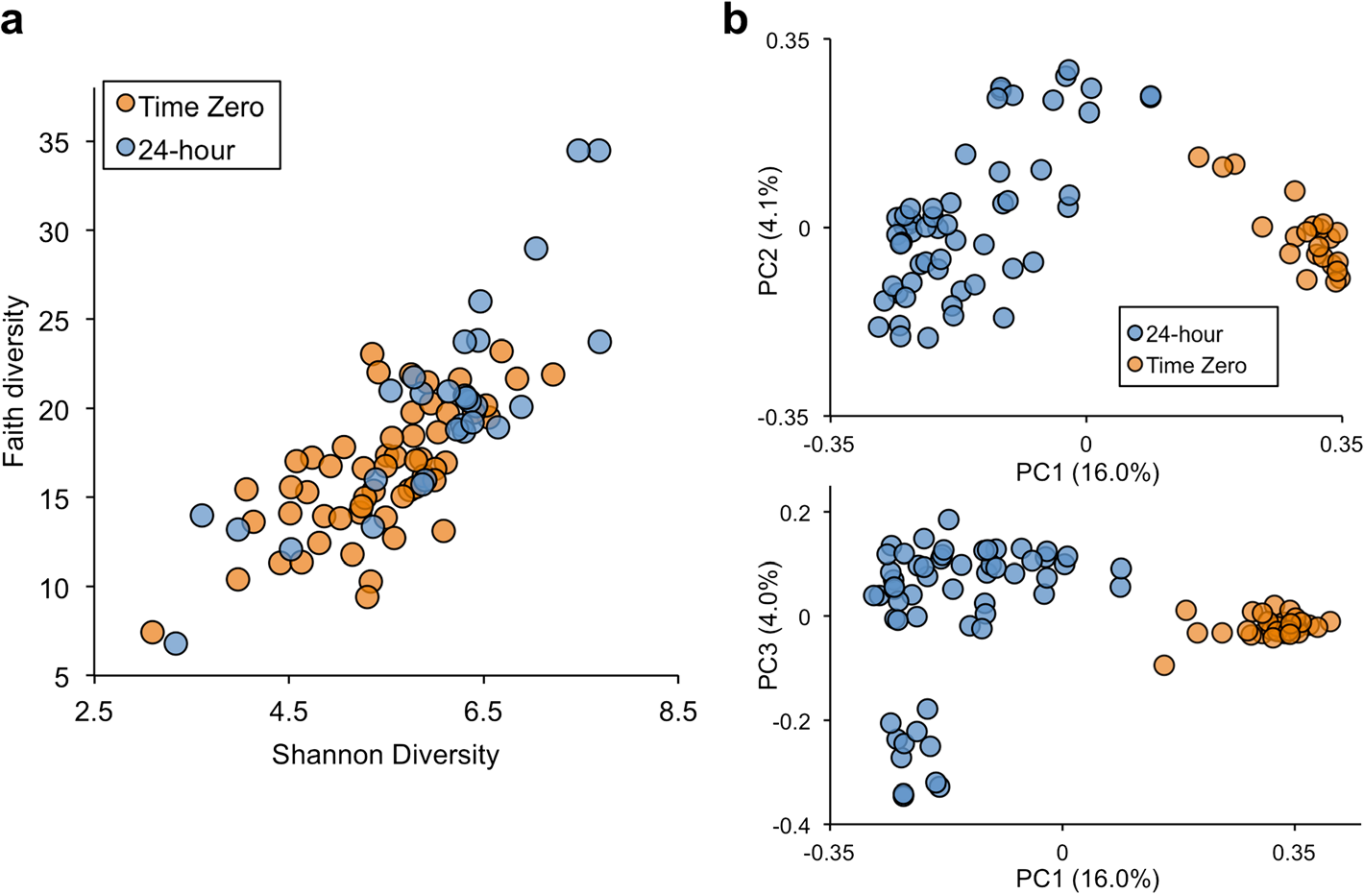
**a** Alpha diversity measured using Shannon entropy and Faith’s whole-tree phylogenetic diversity. Both metrics indicate a significant increase in the diversity of T0 sample communities relative to the T24 group (*P* < 0.002; Mann–Whitney test). **b** Principal coordinates analysis reveals a distinct clustering of samples by T0/T24 status. (PCoA plots computed from unweighted UniFrac distances)

The alpha diversity measured as Shannon entropy and Faith’s whole-tree phylogenetic diversity, showed a significant increase in species richness for T0 sample communities relative to the T24 group (*P* = 0.0007 and *P* = 0.0017, respectively; Mann–Whitney test) (Fig. 3a). We observed no apparent differences in alpha-diversity in samples grouped by known parameters such as state, month, or collection year, nor did we see differences between *Salmonella* culture positive and negative cilantro samples (data not shown). Principal coordinate analysis using a Unifrac distance measure of beta-diversity showed changes to the phylogenetic diversity of cilantro, after the 24-hour nonselective pre-enrichment step, revealed by distinct clusters for the T0 and T24 samples (Fig. 3b) [19]. As with the alpha-diversity, no other sample parameters (state, date of collection) documented in this study gave rise to distinct clustering (data not shown).

### Application of a *Salmonella* detection pipeline

One of the goals of our cilantro study was to assess the efficiency of our initial 24-hour nonselective mBPW pre-enrichment for *Salmonella* species. By default, the QIIME implementation of the RDP classifier uses the GreenGenes database as a training set, which contains only three *S. enterica* reference sequences. Since we did not detect *Salmonella* in our 16S rRNA amplicon data, we sought to improve our assignment specificity by creating a high-quality Enterobacteriaceae specific database (EnteroDB). The EnteroDB, consisting of full-length reference 16S rRNA sequences, including *S. enterica*, and was implemented in BLASTn-based searches for *Salmonella* in our 16S datasets (see Additional file 4).

**Fig. 4:**
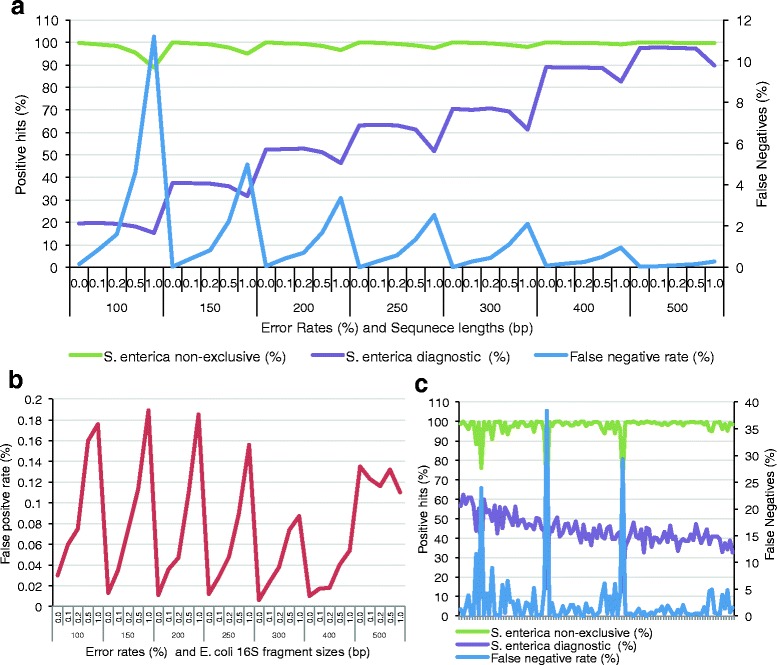
**a** Sensitivity testing of the Enterobacteriaceae database. Randomly fragmented 16S rRNA genes specific to *S. enterica* were compared to the Enterobacteriaceae database using BLASTn. Fragment sizes ranged from 100 to 500 bp and errors were randomly introduced at rates ranging from 0 to 1 %. The *S. enterica* non-exclusive plot (green) represents the percentage of hits to *Salmonella* and other Enterobacteriaceae. The *S. enterica* diagnostic plot (purple) represents the percentage of hits exclusive to *Salmonella* (left axis). The false negative rate plot (blue) represents the percentage of 16S fragments without a *Salmonella* best alignment (right axis). **b** Specificity testing of the Enterobacteriaceae database. 16S rRNA fragments specific to *E. coli* were randomly fragmented to sizes ranging from 100 to 500 bp and random errors were introduced. Fragments were searched against the Enterobacteriaceae database using BLASTn. **c** Validation of the Enterobacteriaceae database using BLASTn analysis of raw Illumina MiSeq reads from 105 *S. enterica* 16S rRNA genes to the EnteroDB. The *S. enterica* non-exclusive plot (green) represents the percentage of hits to *Salmonella* and other Enterobacteriaceae. The *S. enterica* diagnostic plot (purple) represents the percentage of hits exclusive to *Salmonella* (left axis). The false negative rate plot (blue) represents the percentage of 16S rRNA fragments without a *Salmonella* best alignment (right axis)

We performed sensitivity checks of the EnteroDB by simulating variable length *Salmonella* specific 16S rRNA fragments (100–500 bp) with error rates ranging from zero to 1 %. BLASTn analysis revealed a positive correlation between the size of the test fragments (the larger being more sensitive) and the exclusive detection of *S. enterica* (Fig. 4a). Remarkably, the 0 % error rate 500 bp fragments had the highest diagnostic sensitivity of 97.6 % exclusive hits to *S. enterica* (Fig. 4a). As expected the overall non-exclusive hits did not correlate with 16S fragment sizes or error rates since the non-exclusive 16S rRNA fragments match 16S rRNA fragments from *Salmonella* as well as other Enterobacteriaceae (Fig. 4a)*.* As the 16S rRNA fragment sizes increased the false negative rate (number of 16S rRNA fragments without an *S. enterica* best hit) decrease and the lowest false negative rate for all fragment sizes (100–500 bp) was in the 0 % error rate groups, as expected (Fig. 4a). We also observed much lower sensitivities for *S. enterica* using the RDP and UCLUST taxonomic classifiers; simulated 500 bp reads with a 0 % error rate resulted in sensitivities of only 3.82 and 7.19 % respectively [16, 20].

To evaluate the specificity of our EnteroDB, we randomly simulated reads from reference *E. coli* 16S sequences (100–500 bp) with error rates ranging from 0 to 1 %. As expected, the highest false positive rates were associated with the smallest *E. coli* fragments, with all false positive rates remaining below 0.2 %, regardless of the fragment size (Fig. 4b). Interestingly, the false positive rate of the 500 bp fragments remained relatively constant, averaging 0.12 %, regardless of error rate. Further examination identified two *E. coli* 16S rRNA reference sequences closely aligned with *Salmonella* sequences at the 3′ end of the 16S rRNA gene (Fig. 4b).

Validation of the EnteroDB with raw Illumina MiSeq reads of *Salmonella* 16S rRNA fragments from the GenomeTrakr database resulted in diagnostic sensitivities ranging from 38 to 62 %, which increased as the average read length of the *Salmonella* sequences increased (Fig. 4c). Oddly, the false positive rates from three of the 105 genomes tested were very high (24, 38, and 29 %). Further examination of the available metadata from GenomeTrakr revealed that two of the isolates were *S. enterica* subspecies *houtenae* isolated from frozen scad fish, one serovar 44:z4, z32:- from China, the second serovar 43:z4,z23:- from Vietnam, and the third un-typed *S. enterica* isolate originated from frozen cooked snail meat (sea snails) also from Vietnam. Adding the *S. enterica* subspecies *houtenae* genomes to our EnteroDB will expand our diagnostic detection to include these serovars, but as it stands our database has a very high discriminatory power for the detection of *S. enterica* subspecies *enterica* serovars.

Applying EnteroDB to our 16S rRNA T0 and T24 cilantro sample data resulted in only two of T24 samples having exclusive hits to *S. enterica*, one culture negative cilantro sample had a single read hit, likely a false positive. The second, a culture positive *Salmonella* cilantro sample, had 25 hits. We believe the lack of 16S rRNA *Salmonella* hits in the other culture positive cilantro samples resulted from either the low sequencing depth obtained with 454 pyrosequencing, a potential priming bias associated with the 27F primer, because the primer does not perfectly match the *S. enterica* 16S rRNA gene, or a combination of these factors.

### Shotgun metagenomics of cilantro

To investigate the lack of positive hits to *Salmonella* in our 16S rRNA gene libraries, we performed shotgun metagenomic sequencing, on six culture-positive cilantro samples, on the Illumina MiSeq (Table 2). The *Salmonella* isolates cultured from these cilantro enrichments were sequenced, as part of the FDA GenomeTrakr project (http://www.ncbi.nlm.nih.gov/bioproject/186035), and therefore, could be used in BLASTn analyses against the shotgun metagenomes (Table 2). Since no quantitative data were available to estimate *Salmonella* contamination levels in the cilantro samples, three multiplexing strategies were employed using the Illumina 500-cycle v2 chemistry, to evaluate the effect of multiplexing on our ability to detect *Salmonella*.

**Table 2:** Percent hits for BLASTn and MetaPhlAn analysis of cilantro shotgun metagenomes to *Salmonella*

Cilantro sample	FL8K^a^		MI2J^a^		NY7J^a^		MI6F^b,f^		NY3F^c^	OH6F^c,f^		
Time point	T0^d,^	T24^e,^	T0	T24	T0	T24	S2_T24	S3_T24	T24	S1_T24	S2_T24	S3_T24
No. of samples in MiSeq run	1	1	6	6	6	6	2	2	1	6	6	6
Total reads	2,566,734	9,146,885	1,212,903	2,009,022	683,454	1,767,923	5,450,586	4,990,831	10,622,374	3,347,897	2,082,712	1,988,350
BLASTn^g^												
SalC 102 (Newport) (%)	**2.383**	**16.135**	**0.008**	**1.042**	**0.029**	**0.145**	3.959	1.377	0.227	0.128	0.084	0.183
SalC 13 (Newport) (%)	2.347	15.779	0.008	1.016	0.028	0.144	**4.099**	**1.419**	0.228	0.126	0.082	0.183
SalC 77 (Tennessee) (%)	1.785	11.994	0.008	0.788	0.024	0.139	3.061	1.077	**0.215**	**0.110**	**0.074**	**0.214**
SalC AVG (%)	2.172	14.636	0.008	0.949	0.027	0.143	3.706	1.291	0.223	0.121	0.080	0.193
MetaPhlAn												
*S. enterica* (%)	4.870	13.658	0.000	0.000	0.000	0.000	4.282	1.790	0.000	0.000	0.000	0.000
*Salmonella*_unclassified (%)	14.626	34.269	0.000	7.770	0.000	0.000	15.717	6.490	0.000	0.000	0.000	0.448

^a^Cilantro sample culture positive for SalC 102

^b^Cilantro samples culture positive for SalC 13

^c^Cilantro samples culture positive for SalC 77

^d^T0 = time zero

^e^T24 = 24-hour

^f^Subsets of a single cilantro samples are indicated by S1 S2 and S3

^g^Bolded numbers indicate results with S. enterica isolate cultured from the cilantro

MetaPhlAn analysis of cilantro microbiome content, and BLASTn analysis using the *S. enterica* isolates cultured from the cilantro, to estimate the relative abundance of *Salmonella*, revealed variable levels of *Salmonella* contamination in the cilantro metagenomes, even after the 24-hour nonselective mBPW pre-enrichment. For example, two MiSeq runs included single samples, FL8K T0 and FL8K T24, and their metagenomes had the highest relative abundances of *S. enterica* in all of the samples tested, with 2 and 16 % BLASTn hits to the *S. enterica* Newport isolate cultured from the cilantro*,* respectively (Table 2). In contrast, the NY3F T24 pre-enrichment cilantro metagenome contained a much lower number of hits to *Salmonella*, with only 0.215 % of the reads mapping to *S. enterica* Tennessee, despite being a single sample run on the MiSeq. Additionally, the NY3F T24 24-hour pre-enrichment metagenome contained a higher number of sequence reads (10,622,374) than the FL8K T24 24-hour pre-enrichment metagenome (9,146,885) (Table 2). Finally, the BLASTn hits to *S. enterica* Newport in the uncultured sample, FL8K T0, with 2,566,734 reads, are 10 times higher than the BLASTn hits in the 24-hour pre-enrichment cultured sample, NY3F T24, with 10,622,374 reads, corroborating a difference in contamination levels (Table 2).

A third MiSeq run consisted of two T24 samples, MI6F_S2 and MI6F_S3. The BLASTn hits to *S. enterica* in MI6F_S2 and MI6F_S3 were higher than the BLASTn hits in the single sample NY3F T24 run, even though the run consisted of two samples. Additionally, MI6F_S2 and MI6F_S3 both had lower numbers of sequence reads (5,450,586 and 4,990,831) than the single sample NY3F T24 run (10,622,374) (Table 2).

The MI2J, NY7J, and OH6F cilantro samples were multiplexed at six samples per MiSeq run, and, except for MI2J T24, which had 1 % hits to *S. enterica*, the BLASTn percentage hits to *S. enterica* were negligible (Table 2).

The variability in *Salmonella* contamination levels, even after a 24-hour nonselective pre-enrichment, presents a challenge for detecting *Salmonella* amidst the complex background microflora present in cilantro, even in individually sequenced metagenomes. Additionally, multiplexing six samples together appears to have had a negative impact on our ability to detect *Salmonella,* although in one metagenome, MI2J T24, we did identify 1 % of the sequence reads as *S. enterica* Newport (Table 2). It is notable that the MI2J T24 metagenome (2,009,022 reads), had less than half as many reads as MI6F S3_T24 (4,990,831 reads), and yet both metagenomes had ~1 % hits to *S. enterica* supporting our conclusion that sequencing depth and levels of contamination contribute to our ability to detect *Salmonella* in cilantro metagenomes (Table 2).

Analysis of the cilantro shotgun metagenomes using MetaPhlAn, with clade specific marker genes to classify genomic reads, resulted in a similar outcome to the BLASTn analysis, with respect to levels of *S. enterica* (Table 2). The FL8K, MI2J, and MI6F samples returned the highest percentage of hits to *S. enterica* (Table 2). MetaPhlAn also identified unclassified hits to *Salmonella,* representing microbial reads belonging to clades with no sequenced genomes with *Salmonella* as their closest ancestor, at higher percentages than either BLASTn or MetaPhlAn at the species level; a surprising result considering that we used genomes of the isolates cultured directly from the cilantro for the BLASTn analysis (Table 2) [21].

## Discussion

Culture-independent methods have shown the microbial diversity of many plants to be far greater than previously estimated by culture-based methods. 16S rRNA sequencing in particular has revealed Proteobacteria (specifically α- and γ−) as the dominant phyllosphere inhabitants followed by β-Proteobacteria and Firmicutes depending on the type of plant [22–25]. Our results, similar to other 16S rRNA sequencing studies of fresh produce, revealed a potential core phyllosphere for cilantro consisting mainly of Proteobacteria, Bacteroidetes, Actinobacteria, and Firmicutes [26, 27].

Finding that a 24-hour nonselective pre-enrichment of cilantro in mBPW shifts the microbial community to a predominance of Bacillales and Clostridiales, was surprising since our goal is to enrich for Enterobacteriales, specifically *Salmonella*. On average, Bacillales and Clostridiales were 5-fold and 753-fold more abundant in the T24 16S rRNA cilantro samples than in the T0 samples (Table 1). Despite very low frequencies in the initial state, the Bacillales and Clostridiales orders proliferate significantly to represent over 85 % of the T24 16S rRNA reads suggesting that our pre-enrichment process results in a low oxygen environment favoring the resuscitation of Gram-positive bacteria (Table 1).

**Fig. 5:**
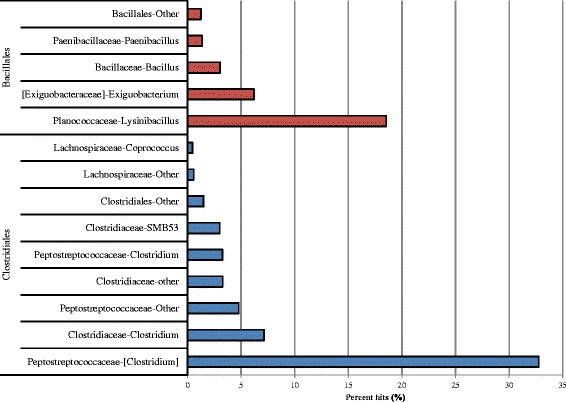
The percentage of hits to members of the Clostridiales and Bacillales orders in cilantro samples enriched for 24-hours in mBPW

Since our enrichment protocol is designed to recover members of the Enterobacteriales, and the Clostridiales showed a relatively high abundance in 24-hour samples, we examined the data at the genus level to determine the predominant members. QIIME identified the majority of *Clostridium* as Peptostreptococcaceae followed by the Clostridiaceae and Lachnospiraceae (Fig. 5). The classification of *Clostridium* has been hampered because historical methods relied on phenotypic characteristics such as Gram-positive staining, anaerobic respiration, and sporulation, to the extent that 16S rRNA taxonomy has exposed the misclassification of 52 % of the species [28–30]. The *Clostridium* species within the Clostridiaceae family represent the genus *Clostridium sensu stricto*, and *Clostridium* species within the Lachnospiraceae and Peptostreptococcaceae have been proposed to fall outside of the *Clostridium* genus [30]. The taxonomic uncertainty in the *Clostridium* species has resulted in the use of brackets to distinguish questionable assignments in the NCBI and SILVA taxonomy databases i.e. [*Clostridium*] [28].

In the 24-hour16S rRNA cilantro samples, the majority of the *Clostridium* OTUs were classified as Peptostreptococcaceae (327) representing 33 % of the total hits to [*Clostridium*] and 3 % (33) to *Clostridium* suggesting a misidentification of taxa within this group (Fig. 5). The remaining *Clostridium* OTUs were distributed between the Clostridiaceae present at (71) 7 % and the Lachnospiraceae present at less than 1 %. The higher proportional abundances of facultative anaerobic Peptostreptococcaceae family members, and lower proportional abundances of Clostridiaceae (mostly obligate anaerobes), matches our expectation of higher abundances of aerobic microorganisms [31]. However, it is noteworthy that 7 % of the taxa represent potentially obligate anaerobes (Fig. 5). A tomato phyllosphere study, using a universal pre-enrichment broth recommended in the BAM method for detection of *Salmonella* in tomato, showed a similar enrichment bias [10]. Overall, this finding suggests that increasing the levels of oxygen in our 24-hour nonselective cilantro pre-enrichments will reduce the Firmicutes taxa and improve the recovery of Proteobacteria.

In this study, we focused first on determining the microbiome of cilantro and then looked at the changes induced by culturing, concentrating on the first step of the FDA BAM method for the detection of *Salmonella* in leafy greens. Soil microorganisms most likely contribute to the diversity observed in the cilantro microbiome since we commonly observed soil contamination in the cilantro samples received. Cilantro is either hand harvested and sold in bundles at retail outlets, or mechanically harvested for distribution in bulk to food service and processing facilities (http://anrcatalog.ucdavis.edu/Details.aspx?itemNo=7236). Hand harvested products are cut just below the soil, or 1.5–2 in. above the crown, secured in bunches by rubber bands or twist ties, packaged in 10 lb boxes, and cooled (0.6–1.7C) at storage facilities, until it is shipped to distribution centers. Mechanically harvested cilantro is conveyed in shallow bins or totes, and is packed in plastic bags of various sizes for use in food service. The point within the distribution process for the cilantro samples used in this study was not available to us. However, the way that they were packaged indicated that, of the 365 samples received, 89 % (326) were packaged with twist ties (14 from Mexico) indicating that they were hand harvested, and 6 % (21) were in restaurant ready plastic bags likely prepared for food service industry. In addition, 2 % (8) of the cilantro samples had organic labels, and 3 % (10) were harvested with roots still attached. We did not rinse or remove soil contamination from the cilantro samples, since they represent the cilantro sold at grocery store, and therefore, can surmise that soil microflora contributed to the higher than expected proportional abundances of Firmicutes in our cilantro data. Studies on the tomato phyllosphere demonstrated a gradient of microbial diversity from the bottom to the top of the plant, with the parts closest to soil having the highest diversity, and Firmicutes significantly co-enriched during an overnight incubation in broth culture [10, 27].

The low abundance of *Salmonella* detection in the cilantro 24-hour pre-enrichments, indicative of very low contamination levels, were unexpected, especially in the culture positive samples, prompting us to create a 16S rRNA database specific to Enterobacteriaceae. The result of only two of the 16S rRNA samples testing positive for *Salmonella* with the EnteroDB was even more unexpected, and likely due to sequence depth limitations inherent to multiplexing 20–22 samples in the 16S rRNA sequencing runs using 454 pyrosequencing. Our laboratory did not have an Illumina MiSeq during the 16S rRNA portion of our cilantro study. However, when the technology became available, we examined a set of cilantro samples using a shotgun metagenomic approach to determine if *Salmonella* detection was possible. The culture based portion of this study, revealing contamination of cilantro with *Salmonella*, was not quantitative, and, therefore, gave us no indication of the concentration of *Salmonella* in the cilantro samples [12]. Overall, our 16S rRNA and shotgun metagenomic findings show the high sensitivity of the FDA BAM culture method for the detection of *Salmonella*, amidst a complex background microflora, and yet in some of the *Salmonella* culture positive metagenomes we were unable to detect *Salmonella* sequences using BLASTn and MetaPhlAn. Additionally, our results indicate that pathogen-spiking studies will add precision to our detection capabilities when comparing 16S rRNA and shotgun metagenomic data for enrichment efficiencies and biases.

Database limitations indicated by the relatively low levels of *Salmonella* detection when implementing the EnteroDB against the RDP or UCLUST taxonomy classifiers, only 3.82 and 7.19 % positive hits respectively, also contribute to false negative results. Therefore, pathogen specific databases will improve taxonomic classification of organisms in complex matrices. The lack of *Salmonella* detection is also due to low primer specificity. The primers used in for 16S rRNA amplification successfully established baseline microbial characterizations in tomato and cheese 16S rRNA surveys, even though the 27F forward primer is missing nucleotide degeneracies commonly used to increase the diversity in the detection capability of PCR [8, 27, 32]. Perhaps incorporating nucleotide degeneracies into our 27F primer would have reduced potential amplification bias in our PCR resulting in higher abundances of Enterobacteriaceae such as *Salmonella* [32].

The factors influencing the survival of animal pathogens such as *S. enterica* in plant and agricultural production environments remain unclear. A number of studies have demonstrated an enhanced fitness of *Salmonella* on cilantro, tomato, and other fruits and vegetables, in the presence of the soft rot producing pathogens such as *Dickeya dadantii,* and *Xanthomonas perforans* [33–35]. Growth rates of *S. enterica* were higher upon co-infection of plant tissues with soft rot producing plant pathogens due to the release of beneficial nutrients [33]. Another study found increased *S. enterica* fitness on cilantro and lettuce leaves when the leaves were pre-colonized with *Pseudomonas syringae* or *Erwinia herbicola,* but not when *S. enterica* alone colonized the leaves, suggesting a mutualistic cohabitation with natural plant epiphytes may be required for long term survival [36]. Considering these findings, we analyzed our T0 16S rRNA data using MetaStats and found evidence of a significant depletion of Gammaproteobacteria in T0 *S. enterica* culture positive samples relative to T0 culture negative samples (*P* = 0. 015) (see Additional file 5). However, since six of the seven T0 *Salmonella* culture positive 16S rRNA libraries contained less than 1000 16S rRNA reads, it is difficult to draw a sound conclusion. Additionally, other taxonomic members of the cilantro phyllosphere and environmental factors may contribute to the fitness and persistence *Salmonella*.

## Conclusion

In conclusion, we have characterized the microbiome of cilantro using 454 pyrosequencing of 16S rRNA genes and described the transition of the microbial community after a 24-hour nonselective pre-enrichment step. Our approach has revealed a substantial shift in microbial community structure during a 24-hour nonselective pre-enrichment step that seems to favor members of the Firmicutes. Whole genome shotgun metagenomic analysis of *Salmonella* culture positive cilantro samples revealed variable levels of *Salmonella* contamination emphasizing the need for controlled spike studies in order to predict the shifts in abundances of *Salmonella* during the nonselective and selective enrichment steps used in the FDA BAM. Future work to improve the utility of 16S rRNA and shotgun metagenomic sequencing, and analysis, for the detection of *Salmonella* are underway. Considering the importance of identifying foodborne pathogens during outbreaks, this study demonstrates the use of high-throughput sequencing to understand the enrichment and identification pathogens in a leafy green commodity.

## Methods

### Cilantro sample collection

Cilantro samples were processed using a modified FDA BAM method. Briefly 100 g samples of cilantro were aseptically combined with 500 ml of mBPW in sterile whirlpak bags (Nasco, Fort Atkinson, WI), gently massaged for 2 min and a 1 ml aliquot was removed prior to and after a 24-hour static incubation at 37 °C. Nine cilantro samples were culture positive for *Salmonella*. Genomic DNA was prepared using the Nuclisens® easyMAG® with the following conditions: 1.0 ml aliquots were processed using Protocol B, D 2.0.0, with an elution volume of 70 μl (Biomerieux, Durham, NC).

### 16S rRNA sequencing

16S rRNA amplicon sequencing on the Roche GS FLX Titanium 454 pyrosequencing platform (454 Life Sciences, a Roche company, Branford, CT 06405) was performed on 91 samples. Amplicons spanning the V1-V3 regions of the 16S rRNA gene, were generated with Roche Fusion Primer A 27F (5′ CGT ATC GCC TCC CTC GCG CCA TCAG ACG AGT GCG T AGA GTT TGA TCC TGG CTC AG 3′), with MIDs (multiplex identifiers) 1 – 22, and Roche Fusion Primer B, 533R (5′ CTA TGC GCC TTG CCA GCC CGC TCAG TTA CCG CGG CTG CTG GCA C 3′), with no MIDS. Emulsion PCR of amplicon libraries was carried out using the Lib-A MV kit (Roche) and samples were multiplexed (20–22 samples) in five medium regions of a PicoTiterPlate.

### Whole genome shotgun sequencing

Shotgun metagenomes of cilantro were prepared using the Illumina Nextera sample processing kit and sequenced on a MiSeq (Illumina Inc., San Diego, CA). Briefly, 50 ng of genomic DNA were fragmented and tagmented and unique indexes were added using reduced-cycle PCR amplification. Amplicon libraries were size selected using Agencourt AMPure XP beads (Beckman Coulter, Brea, CA) to an average library size of 500 bp, and quantified using the Qubit 3.0 Fluorometer (Life Technologies, Carlsbad, CA). Library quality was verified on the Agilent Technologies 2100 Bioanalyzer (Agilent Technologies, Inc., Santa Clara, CA) using the High Sensitivity DNA chip kit. Size selected libraries were normalized to 2nM, pooled in equal volumes and run on a 500-cycle MiSeq Reagent Kit v2 (Illumina Inc., San Diego, CA). Seven of the metagenomes (MI2J T0, MI2J T24, NY7J T0, NY7J T24, OH6F S1_T24, OH6F S2_T24, and OH6F S3_T24) along with five samples that were not part of this study, were multiplexed into two MiSeq runs, consisting of six samples each (Table 2). Two metagenomes (MI6F S2_T24 and MI6F S3_T24) were sequenced together in a single MiSeq run, and the remaining three metagenomes (FL8K T0, FL8K T24, and NY3F T24) were sequenced individually (Table 2).

### 16S sequence analysis

Raw reads generated by the Roche/454 platform were initially filtered for length (≥150 bp) and quality using the QIIME platform, requiring a maximum homopolymer run of 8 nucleotides and an average Phred quality score of 25 [13]. The resulting high-quality sequence set was also trimmed of forward and reverse primer sequences and split by sample membership according to 5′ multiplex identifiers (MIDs). Sequences were then screened for chimeras using USEARCH (*de novo* mode) [20, 37], and subsequently assessed for contaminant chloroplast sequences with the RDP classifier [16–18, 20, 37].

Passing sequences were clustered into Operational Taxonomic Units (OTUs) (*de novo*) by UCLUST using a 97 % similarity threshold [20]. Representative sequences (defined as the most abundant sequence) from each OTU were assigned a taxonomic lineage using the RDP classifier [16] trained on the GreenGenes 16S rRNA database (v13_8) with a minimum threshold of 0.50 [17, 18]. Representatives were further aligned to a template multiple sequence alignment using PYNAST [38], which was then filtered for columns with excessive gap content and used to construct a phylogenetic tree with FastTree2 [38, 39].

The resulting OTUs were then evaluated for alpha and beta-diversity in QIIME. Alpha diversity measures included Shannon entropy and Faith’s whole-tree diversity. OTU counts were rarefied to 1000 sequences per sample prior to downstream statistical analysis. MetaStats (set to 5000 permutations for the nonparametric *t*-test) was employed for differential abundance analysis, and p-values were adjusted using the false discovery rate (FDR) [15].

### MiSeq shotgun sequence analysis

Raw whole genome shotgun paired reads were quality filtered and trimmed using CLC bio Workbench v6.0.4 (CLC Bio, QIAGEN, Germantown, MD) with an average Phred score of 25, allowing two ambiguities per read and removing any read less than 75 bp. Trimmed reads were merged with a mismatch cost of 2, a gap cost of 3, and a minimum score of 8. The merged (read 1/read 2) Illumina dataset was then combined with the read 1 sequences that passed the quality filtering, but did not merge with their read 2 paired sequence. High-quality sequences were analyzed for clade specific marker genes using Metaphlan [21] with Bowtie2 for alignments (‘very-sensitive’ mode) [21, 40]. For improved sensitivity, the nucleotide sequences were also searched using the (BLASTn) for high-identity alignments to a custom database of *S. enterica* strains isolated from cilantro samples. Alignments to *S. enterica* with at least 98 % identity and at least 95 % coverage of the query sequence were aggregated per sample.

### *Salmonella* detection pipeline

To perform species-level assignments of 16S rRNA sequences to *S. enterica*, we first developed a comprehensive database of all Enterobacteriaceae species available in the Silva database (v111) [41]. Multiple rounds of clustering and refinement were performed to identify poor quality sequences and mis-annotated species. The database was augmented with 25 additional *S. enterica* reference 16S rRNA sequences from cultured isolates associated with cilantro. A final round of refinement was performed through phylogenetic analysis of all sequences (MUSCLE for multiple alignments, FastTree2 for tree construction) [39, 42].

The final database contains 2804 sequences from 189 unique species within Enterobacteriaceae, including 144 from *S. enterica* reflecting 28 unique serovars. Using this database, we designed an efficient *Salmonella* detection pipeline based on the *parallel_blast.py* tool implemented in QIIME [13]. The pipeline performs local heuristic alignment of 16S rRNA amplicon sequences to the database and collates all alignments with the highest overall bit score. Sequences exclusively matching *S. enterica* with at least 98.5 % identity along at least 97 % of their length are classified as *S. enterica*.

We evaluated our sensitivity and specificity by simulating noisy reads from *S. enterica* and *E. coli* 16S rRNA genes with variable lengths (100–500 bp) and error rates up to 1 %. All typed reference sequences available in the RDP 16S rRNA database (*n* = 9244) were also extracted to test for sensitivity/specificity [43]. We further validated our approach using raw Illumina MiSeq 16S rRNA sequence data from 105 *S. enterica* isolates deposited by the FDA GenomeTrakr project. Validation of the sensitivity and specificity of the Enterobacteriaceae Database (EnteroDB) was implemented by BLASTn comparison against a set of raw 16S rRNA sequence reads extracted from the FDA GenomeTrakr database (http://www.ncbi.nlm.nih.gov/bioproject/183844) specific to *Salmonella*. Bowtie2 was employed to extract 16S rRNA fragments from each sample as a positive control dataset [40]. For comparison of assignment performance, the RDP classifier and UCLUST_ref were run using default settings in QIIME [13, 16].

### Sequencing data

The raw 454 and shotgun metagenomic data are uploaded to NCBI SRA. The Bioproject accession number is PRJNA260637. The EnteroDB is available upon request.

## Additional files

Additional file 1:
**Metadata for cilantro samples.** (XLSX 14 kb) Additional file 2:
**Preprocessing results of 16S rRNA sequences.** (XLSX 15 kb) Additional file 3:
**Genus level abundances of Enterbacteriaceae.** (XLSX 64 kb) Additional file 4:
**Enterobacteriaceae database files.** (ZIP 132 kb) Additional file 5:
**High abundance classes among T0 samples.** (XLSX 10 kb)
